# Supplementary material for: Persistence of Multiple Paramyxoviruses in a Closed Captive Colony of Fruit Bats (Eidolon helvum)
Source: Viruses. 2021 Aug 20;13(8):1659. doi: 10.3390/v13081659 (PMC8402880; doi:10.3390/v13081659)
Supplement: Supplementary file 1 [file viruses-13-01659-s001.zip › viruses-1326198-supplementary.pdf]

SUPPLEMENTARY MATERIAL

Table S1. Top BLAST results from submission of the consensus sequence of the L gene. Abbreviations: P = 530 bp fragment obtained with the pan-*Paramyxovirus* RT-PCR, R = 439 bp fragment obtained with the *Respiro-Morbilli-Henipavirus* RT-PCR.

Scheme .	Number of detections	GenBank BLAST ID	GenBank Accession	Nucleotide identity (%)
**Z15-U17P**	1	Bat Paramyxovirus Eid_hel/ZFB09-07/Zambia/2009 L gene for RNA polymerase, partial cds	AB853094.1	99.25
**Z15-U27P**	15	Eidolon helvum paramyxovirus clone U68G large protein (L) gene, partial cds	JN648082.1	96.98
		Eidolon helvum paramyxovirus clone U64A large protein (L) gene, partial cds	JN648077.1	96.79
**Z15-U111P**	1	Eidolon helvum paramyxovirus clone U69D large protein (L) gene, partial cds	JN648084.1	98.87
		Eidolon helvum paramyxovirus clone U69C large protein (L) gene, partial cds	JN648083.1	98.68
**Z15-U12R**	31	Paramyxovirus bat/GH27a/2009 polymerase (L) gene, partial cds	FJ971940.1	98.63
		Eidolon helvum paramyxovirus clone U61A polymerase (L) gene, partial cds	JN862578.1	98.38
**Z15-U17R**	3	Bat Paramyxovirus Eid_hel/GB1678/GAB/2005 isolate BatPV/Eid_hel/GB1678/GAB/2005 RNA-dependent RNA polymerase (L) gene, partial cds	HQ660144.1	99.54
		Bat Paramyxovirus Eid_hel/GB1535/GAB/2005 isolate BatPV/Eid_hel/GB1535/GAB/2005 RNA-dependent RNA polymerase (L) gene, partial cds	HQ660141.1	99.08
**Z15_U27R**	11	Eidolon helvum paramyxovirus clone U51B polymerase (L) gene, partial cds	JN862573.1	99.54
		Eidolon helvum paramyxovirus clone U51A polymerase (L) gene, partial cds	JN862564.1	99.09
		Eidolon helvum paramyxovirus clone U50B polymerase (L) gene, partial cds	JN862580.1	98.18
		Eidolon helvum paramyxovirus clone U63A polymerase (L) gene, partial cds	JN862574.1	98.18
		Eidolon helvum paramyxovirus clone U64A polymerase (L) gene, partial cds	JN862575.1	97.72
		Eidolon helvum paramyxovirus clone U49B polymerase (L) gene, partial cds	JN862572.1	97.72
**Z15-U78R**	13	Bat paramyxovirus Eid.hel/GH45/2008 polymerase (L) gene, partial cds	GQ168929.1	99.09
		Bat Paramyxovirus Eid_hel/GH-M61a/GHA/2009 isolate BatPV/Eid_hel/GH-M61a/GHA/2009 RNA-dependent RNA polymerase (L) gene, partial cds	HQ660133.1	95.67
		Eidolon paramyxovirus RC09 partial L gene for RNA-dependent RNA polymerase, isolate EPMV_RC09_214s	HE647822.1	94.99
**Z15-U86R**	10	Eidolon helvum paramyxovirus clone U54B polymerase (L) gene, partial cds	JN862571.1	97.72
		Eidolon paramyxovirus RC09 partial L gene for RNA-dependent RNA polymerase, isolate EPMV_RC09_222k	HE647825.1	97.49
		Eidolon helvum paramyxovirus clone U54A polymerase (L) gene, partial cds	JN862563.1	97.49
		Eidolon helvum paramyxovirus clone U59B polymerase (L) gene, partial cds	JN862579.1	97.27

**Z15-U115R**	1	Eidolon helvum paramyxovirus clone U58B polymerase (L) gene, partial cds	JN862583.1	76.26
		Eidolon helvum paramyxovirus clone U32A polymerase (L) gene, partial cds	JN862594.1	75.8

Table S2. Information of the virus sequences used for phylogenetic analyses downloaded from GenBank (NCBI).

Abbreviated as displayed on tree	GenBank Accession	GenBank ID
**Measles virus**	AB016162.1	Measles virus genomic RNA, complete sequence
**BatPV Eid hel ZFB09 07 Zambia**	AB853094.1	Bat Paramyxovirus Eid_hel/ZFB09-07/Zambia/2009 L gene for RNA polymerase, partial cds
**Canine distemper virus**	AF014953.1	Canine distemper virus, complete genome
**Hendra virus**	AF017149.3	Hendra virus, complete genome
**Tupaia paramyxovirus**	AF079780.2	Tupaia paramyxovirus, complete genome
**Newcastle disease virus**	AY845400.2	Newcastle disease virus strain LaSota, complete genome
**J-virus**	AY900001.1	J-virus, complete genome
**Nipah virus Bangladesh**	AY988601.1	Nipah virus from Bangladesh, complete genome
**BatPV GH27a Ghana**	FJ971940.1	Paramyxovirus bat/GH27a/2009 polymerase (L) gene, partial cds
**BatPV Eid hel GH45**	GQ168929.1	Bat paramyxovirus Eid.hel/GH45/2008 polymerase (L) gene, partial cds
**Tuhoko virus 1**	GU128080.1	Tuhoko virus 1, complete genome
**Tuhoko virus 2**	GU128081.1	Tuhoko virus 2, complete genome
**Tuhoko virus 3**	GU128082.1	Tuhoko virus 3, complete genome
**Eidolon PV isolate EPMV RC09 214s**	HE647822.1	Eidolon paramyxovirus RC09 partial L gene for RNA-dependent RNA polymerase, isolate EPMV_RC09_214s
**Eidolon PV isolate EPMV_RC09_222kl**	HE647825.1	Eidolon paramyxovirus RC09 partial L gene for RNA-dependent RNA polymerase, isolate EPMV_RC09_222k
**Ghanaian bat henipavirus**	HQ660129.1	Bat Paramyxovirus Eid_hel/GH-M74a/GHA/2009, complete genom
**BatPV Eid hel M61a Ghana**	HQ660133.1	Bat Paramyxovirus Eid_hel/GH-M61a/GHA/2009 isolate BatPV/Eid_hel/GH-M61a/GHA/2009 RNA-dependent RNA polymerase (L) gene, partial cds
**BatPV Eid hel GB1535 Gabon**	HQ660141.1	Bat Paramyxovirus Eid_hel/GB1535/GAB/2005 isolate BatPV/Eid_hel/GB1535/GAB/2005 RNA-dependent RNA polymerase (L) gene, partial cds
**BatPV Eid hel GB1678 Gabon**	HQ660144.1	Bat Paramyxovirus Eid_hel/GB1678/GAB/2005 isolate BatPV/Eid_hel/GB1678/GAB/2005 RNA-dependent RNA polymerase (L) gene, partial cds
**BatPV Eid hel clone U44A**	JN648059.1	Eidolon helvum paramyxovirus clone U44A large protein (L) gene, partial cds
**BatPV Eid hel clone U49B**	JN648067.1	Eidolon helvum paramyxovirus clone U49B large protein (L) gene, partial cds
**BatPV Eid hel clone U64A**	JN648077.1	Eidolon helvum paramyxovirus clone U64A large protein (L) gene, partial cds
**BatPV Eid hel clone U66A**	JN648078.1	Eidolon helvum paramyxovirus clone U66A large protein (L) gene, partial cds

**BatPV Eid hel clone U68G**	JN648082.1	Eidolon helvum paramyxovirus clone U68G large protein (L) gene, partial cds
**BatPV Eid hel clone U69C**	JN648083.1	Eidolon helvum paramyxovirus clone U69C large protein (L) gene, partial cds
**BatPV Eid hel clone U69D**	JN648084.1	Eidolon helvum paramyxovirus clone U69D large protein (L) gene, partial cds
**BatPV Eid hel clone U6B**	JN648086.1	Eidolon helvum paramyxovirus clone U6B large protein (L) gene, partial cds
**BatPV Eid hel clone U9D**	JN648089.1	Eidolon helvum paramyxovirus clone U9D large protein (L) gene, partial cds
**BatPV Eid hel clone U6B**	JN862562.1	Eidolon helvum paramyxovirus clone U6B polymerase (L) gene, partial cds
**Bat PV Eid hel clone U54A**	JN862563.1	Eidolon helvum paramyxovirus clone U54A polymerase (L) gene, partial cds
**Bat PV Eid hel clone U51A**	JN862564.1	Eidolon helvum paramyxovirus clone U51A polymerase (L) gene, partial cds
**BatPV Eid hel clone U62A**	JN862567.1	Eidolon helvum paramyxovirus clone U62A polymerase (L) gene, partial cds
**BatPV Eid hel clone U59A**	JN862570.1	Eidolon helvum paramyxovirus clone U59A polymerase (L) gene, partial cds
**Bat PV Eid hel clone U54B**	JN862571.1	Eidolon helvum paramyxovirus clone U54B polymerase (L) gene, partial cds
**Bat PV Eid hel clone U51B**	JN862573.1	Eidolon helvum paramyxovirus clone U51B polymerase (L) gene, partial cds
**Bat PV Eid hel clone U63A**	JN862574.1	Eidolon helvum paramyxovirus clone U63A polymerase (L) gene, partial cds
**Bat PV Eid hel clone U64A**	JN862575.1	Eidolon helvum paramyxovirus clone U64A polymerase (L) gene, partial cds
**BatPV Eid hel clone U42B**	JN862577.1	Eidolon helvum paramyxovirus clone U42B polymerase (L) gene, partial cds
**Bat PV Eid hel clone U61A**	JN862578.1	Eidolon helvum paramyxovirus clone U61A polymerase (L) gene, partial cds
**Bat PV Eid hel clone U59B**	JN862579.1	Eidolon helvum paramyxovirus clone U59B polymerase (L) gene, partial cds
**Bat PV Eid hel clone U50B**	JN862580.1	Eidolon helvum paramyxovirus clone U50B polymerase (L) gene, partial cds
**Bat PV Eid hel clone U58B**	JN862583.1	Eidolon helvum paramyxovirus clone U58B polymerase (L) gene, partial cds
**BatPV Eid hel clone U68B**	JN862590.1	Eidolon helvum paramyxovirus clone U68B polymerase (L) gene, partial cds
**Bat PV Eid hel clone U32A**	JN862594.1	Eidolon helvum paramyxovirus clone U32A polymerase (L) gene, partial cds
**Achimota pararubulavirus 1**	JX051319.1	Achimota virus 1, complete genome
**Mojiang virus**	KF278639.1	Mojiang virus isolate Tongguan1, complete genome
**Hendra virus Horse**	MN062017.1	Hendra henipavirus strain HeV/Australia/1994/Horse18, complete genome
**Achimota pararubulavirus 3**	MT062420.1	Achimota pararubulavirus 3 isolate U72, complete genome
**Sendai virus**	NC_001552.1	Sendai virus genomic RNA, antisense, complete sequence
**Human parainfluenza virus 3**	NC_001796.2	Human parainfluenza virus 3, complete genome

**Mumps virus**	NC_002200.1	Mumps virus, complete genome
**Nipah virus Malaysia**	NC_002728.1	Nipah virus, complete genome
**Human rubulavirus 2**	NC_003443.1	Human rubulavirus 2, complete genome
**Human parainfluenza virus 1**	NC_003461.1	Human parainfluenza virus 1, complete genome
**Tioman virus**	NC_004074.1	Tioman virus, complete genome
**Mossman virus**	NC_005339.1	Mossman virus, complete genome
**Rinderpest virus**	NC_006296.2	Rinderpest virus (strain Kabete O), complete genome
**Peste des petits ruminants virus**	NC_006383.2	Peste des petits ruminants virus complete genome
**Simian virus 41**	NC_006428.1	Simian virus 41, complete genome
**Parainfluenza virus 5**	NC_006430.1	Parainfluenza virus 5, complete genome
**Beilong virus**	NC_007803.1	Beilong virus, complete genome
**Mapuera virus**	NC_009489.1	Mapuera virus, complete genome
**Porcine rubulavirus**	NC_009640.1	Porcine rubulavirus, complete genome
**Nariva virus**	NC_017937.1	Nariva virus, complete genome
**Human parainfluenza virus 4**	NC_021928.1	Human parainfluenza virus 4a viral cRNA, complete genome, strain: M-25
**Sosuga virus**	NC_025343.1	Sosuga virus isolate 2012, complete genome
**Cedar virus**	NC_025351.1	Cedar virus isolate CG1a, complete genome
**Achimota pararubulavirus 2**	NC_025404.1	Achimota virus 2, complete genome
**Bat mumps orthorubulavirus**	NC_038271.1	Bat Paramyxovirus Epo_spe/AR1/DRC/2009, complete genome
**Menangle virus**	NC_039197.1	Menangle virus isolate Australia/bat/2009/Cedar Grove, complete genome

Table S3. Nucleotide sequences detected using PCR.

Abbreviations: P = 530 bp fragment obtained with the pan-*Paramyxovirinae* RT-PCR, R = 439 bp fragment obtained with the *Respiro-Morbilli-Henipavirus* RT-PCR

Sequence Name	Genbank Accession ID: description	Nucleotide Sequence
**Z15-U12R**	MZ393364<br>RMH Bat Eid hel/GH/2015/U12R	ATATCGAGACAGACATGGTGGAATTTGGCCAGAATGTTATCTCCCCAACCAC-<br>GCTTCTCAAATAATTAAGAGTAAAAAGATGAATGGGGAAGGGTTATCTATAGAG-<br>GATTGTGTCGAAAACTGGCGATCATTTTGTGGAT-<br>TTAAATTCGGGTGTTTTATGTCTTTAACTTTAGATGAGGATCTGAGCATGTACATGAAAGA-<br>CAAAGCTTTGTCTCCCATTAAATCACAATGG-<br>GACTCTGTTTACCCTAATGCCAACATGAAATACACGCCAAAGCCTAGTACTA-<br>CATCCAGAAGATTGGTAGACGTATTTATCAATGATGCTGAGTTTGAACCGTCAAATTTAA-<br>TAAACTATGTAATCAACGGTGACTATTTGGTGGATGAAGAT-<br>TTCAACATTTCTTATAGTCTAAAAGAAAAAGAAGTCAAAGAAGTTGGGAGACTG
**Z15-U17R**	MZ393365<br>RMH Bat Eid hel/GH/2015/U17R	TTATAGAGACAGGCATGGAGGTGCCTGGCCTCCCGTCAAGTTTCCAGAGCAC-<br>TGCTCAAAAA-<br>GCATACTGAGACTCAAAAATTCTGGCGAATCTATTACTATAGAAGATTGTGTTAAAAACTG<br>GGAATCATTTTGCGGCATTGAATTCGGTTGTTTTATGGACTTAAACCTGGACAC-<br>TGATCTAA-<br>GCATGTATATGAAAGATAAGGCTTTGTCACCTATCAAAGAAGAATGGGATAGTGTATACC<br>CTAAAGAGGTATTGTCATATAAACCTCCCAAATTAACAGAGCCCAGAAGACTAG-<br>TAGATGTATTTGTCAATGACCCAGATTTTGATCCTTATGAAATGATTCAGTATGTCTTGACA

		GGAGCATACCTAACAGACCCGAAGTTCAATGTCTCGTATAGTTTGAAAGAGAAA-<br>GAAACTAAGCAAGCAGGGAGATTA
**Z15-U27R**	MZ393366<br>RMH Bat Eid hel/GH/2015/U27R	TTTTAGAGATCAACATGGAGGTGCTTGGCCTCCGGTAGTTTTAC-<br>CAAATCATGCAGACCCTAGGATAAAAAGACTTAAAAACAATGATGAAGCAATCTC-<br>TATTGAGGATGCCATTAAATACTGGAAATCATTTGTAGGGTTCCACTTTAAGA-<br>CATTTATGCCTTTAAACTTGGACAAAGATTTAAGTATGTATATGAAAGA-<br>CAAGGCTTTATCACCAATCAAATCAGAGTGGGATTCTGTATACCCAAAAGATAA-<br>TATGCTTTATGTTCCTCCGAAAAATACAACATCAAGAAGATTAGTTGATGTATTCCTT-<br>GATGATTCCGATTTTGATCCTATGAATTTGATCAATTATGTTATATCTGGTGATTATTT-<br>GAAAGATCCTGATTTCAACATATCTTATAGTTTAAAGGAAAAAGAGACAAAACAAGTT-<br>GGTCGGTTA
**Z15-U78R**	MZ393367<br>RMH Bat Eid hel/GH/2015/U78R	GTATAGAGATAGACATGGAGGTACTTGGCCACCCTGTAATCTTCCTCCCCATTGTTCAC-<br>CTGCCATTCGAAAAAAACAACTAAATGCAGAATCAGTGTCAATAGAGGACTG-<br>TATTCAAAATTGGCAATCATTTTGTGGGTTCAGTTTCAAGTGTTTTATGCCATTATCATT-<br>AGATTCAGATCTTAGTATGTACATGAAGGACAAAGCATTATCACCTAA-<br>TAAATCAGAATGGGACTCTGTCTATCCCGCAACAAACACCAAATACACACCACCTA-<br>GATCTCAAACATCTAGGAGATTAGTTGATAGGTTCATTGAAGATGATGAGTTTGAAC-<br>CATCTGACATGATCAATTATGTCCTTTCAGGTGAGTATCTCAGTGATGATACATTTAA-<br>TATCTCATACAGTCTAAAAGAGAAAGAAGTCAAAGAAGTCGGACGATTA
**Z15-U86R**	MZ393368<br>RMH Bat Eid hel/GH/2015/U86R	CTATCGAGATCAACATGGAGGTGCCTGGCCTCCTGTTCACTTCAAGGACCATATAA-<br>GCAAAAAGATCAAGAGGTTAATGATTAATAATGAGGCAATATCTGTAGAAGATGCAG-<br>TTCTGAACTGGAAATCCTTTATAGGTTTCAAATTTGGTATATTTATGCCACTTGATCTGGA-<br>TAGTGATTTGAGCATGTATATGAAAGATAAGGCATTATCTCCATTGAACAAAGATTGG-<br>GATTCCGTTTATCCTAAGGAAAATTTGAAATACACACCCCCGAAAAGCCAAGTCTCAA-<br>GACGTCTCGTGGATGTATTTTTAGAAGATGAACAATTTGACCCTGTCAATTTGATTAAC-<br>TATGTTCTTAGTGGAGAATACTTACAAGACAAGAATTTCAACATCTCTTATAGTTTAAAA-<br>GAAAAGGAGACAAAACAAGCTGGTCGACTA
**Z15-U115R**	MZ393369<br>RMH Bat Eid hel/GH/2015/U115R	ATTCCGAGATCAACATGGAGGTGCTTGGCCCCCTATCACATTCCCAGAGCATGTTGA-<br>TAAACATATTAAAAGACTACAAATCAATAGCGAAGCAATATCTGTAGAAGATGCTATACT-<br>AAATTGGAGATCTTTTGTTGGGTGCCATTTCAAGCAATTTATGCCTCTCGATTTAGA-<br>TAAGGATCTTAGTATGTACATGAAAGATAAAGCATTATCCCCAATTAAAAAGGAATGG-<br>GATTCAGTATATCCTAGGGACAGTATGTATTATTGTCCTCCCAAATCAACGACTTCAC-<br>GCAGATTGGTTGAGGTATTCTTAGAAGATTCTGAATTCGATCCAGCAGAACTAATGGAG-<br>TATGTATTGTCTGGCGATTATCTTAATGACCAAGATTTTAATATAAGTTACAGTCTCAAA-<br>GAGAAAGAAACAAAACAAGTGGGTAGACTT
**Z15-U17P**	MZ393370<br>PAR Bat Eid hel/GH/2015/U17P	TGAATCAATAGCGATCACACAGAAAGTTCATCCAAATTTAC-<br>CATACAGGGTCAAAAAGGAAATATGTGCAAAGCAAGCACAAATATATTTCAAGAGAT-<br>TAAGGGAAAATTTAAGATCATTAGGCCATGATCTCAAGGCAACTGAAACAATTATCAG-<br>CACACACCTCTTTGTATACTCCAAGAAAATACATTATGATGGGGCAGTACTTTCCCAA-<br>GCACTTAAATCCATATCTAGGTGTTGTTTCTGGTCAGAGACATTAGTG-<br>GATGAAACTAGGTCAGCATGCAGTAATATCAGCACTGCTATAGCTAAAAGCATAGA-<br>GAATGGTCTATCTAAAGATGTAGGCTACTGCTT-<br>GAACTTTTTAAAAGTTTTACAACAATTACTGATTGCGACTGAATTCGGAATTAATGATAC-<br>GTTGACACCAGACGTTACCAGGCCGATAACTGAAAACCCGGAGTGGTTAATTACAGCGG-<br>CATTAATCCCAGCACCAATTGGAGGATACAACTATTTGAATATGTCTAGAATATACGTTA
**Z15-U27P**	MZ393371<br>PAR Bat Eid hel/GH/2015/U27P	CCAATCAATAGCAATCACAAGGAGAGTTCATCCCTCATTAAGTTTCAAAGCCAAAAAAGA-<br>TATTGCTGCAGATACAACACAAAAATACTTCTTAAGACTTCGGGAGAATCTAAGAGCTTTA<br>GGTCATGATCTTAAAGCAAACGAAACAATTATGAGCACAGCATTTTTTGTATATTCAAA-<br>GAA-<br>GATATATTATGATGGACTTGTATTGTCACAGGCACTTAAATCTATTGCAAGATGTTGCTTTT<br>GGTCTGAAACACTAGTGGATGAATCACGATCAGCATGTTCAAACATATCAACTACCATT-<br>GCCAAATCTATTGAAAACGGATTATCGAAGAAAGCAGGTTACTGCCTTAATCTACTAAAAA

		CTCTTCAACAATTATACATTACCCTGGGATTCAATATTAATGAGACATTAACAGAG-GATGTGACTAAATCTATGATCCAAAATTATAGCTGGTTGATATCAGCTTCATTACTTCCAGC TCCTTTAGGAGGATTCAATTATCTTAATATGTCCCGTGTGTTTGTAA
**Z15-U111P**	MZ393372<br>PAR Bat Eidhel/GH/2015/U111P	TCAAGCTATCGCAATTACTGCAAAGGTGCCTCGTGCAGCTCCGTACCAAGAGAAGAAGA-GAATAGCATTTGAGAATAGCAAATTGTTCATTGAGCGTCTTAGAGCAAATAA-TAAAGGATTAGGACATCATCTCAAAGAGCAAGAGACAATTTTGAGTTCCGAGTTCTTT-GTTTACAGCAAGCGCATCTTTTTTCGCGGAAGGAT-TTTAAATCAGGCATTAAAAAATGTTAGCAAACTAAACCTAATT-GCAGATGTTCTAAGTGAATGCAGTCAGACTTCATGTGCTAATTTGACCACAACAG-TGATGAGACTCACAGAAAATGGGGTCGAGAAAGATATTTGTTACTTCCTCAG-TATTTATCTCTCTATTAAGCAATTGGTCTATGATCTGATGTTCCCGATGACTACATTCATT-GAAGATGCTATTACTTCTTTATATCTCAACCACCCTATCTTGATTGCTAGAC-TATGTGTGTTACCTTCACAGCTTGGCGGGCTCAATAATTACTCAATCAGCAGAT-TATTTAACA
